# An evaluation of the effectiveness of a multi-modal intervention in frail and pre-frail older people with type 2 diabetes - the MID-Frail study: study protocol for a randomised controlled trial

**DOI:** 10.1186/1745-6215-15-34

**Published:** 2014-01-24

**Authors:** Leocadio Rodríguez-Mañas, Antony J Bayer, Mark Kelly, Andrej Zeyfang, Mikel Izquierdo, Olga Laosa, Timothy C Hardman, Alan J Sinclair

**Affiliations:** 1 Division of Geriatrics, Hospital Universitario de Getafe, Carretera de Toledo Km 12.5, 28905, Getafe, Spain; 2 Fundación para la Investigación Biomédica, Hospital Universitario de Getafe, Carretera de Toledo Km 12.5 28905, Getafe, Spain; 3 Institute of Primary Care and Public Health, School of Medicine, Cardiff University and Memory Team, Cardiff and Vale University Health Board, Cardiff CF14 4YS, UK; 4 Institute of Translation, Innovation, Methodology and Engagement (TIME), South East Wales Trials Unit, School of Medicine, Cardiff University, Cardiff CF14 4XN, UK; 5 University of Ulm, Institue of Epidemiology, Albert-Einstein-Allee 41, 89081 Ulm, Germany; 6 Department of Health Sciences, Public University of Navarre, Campus of Tudela 31500 Tudela, Spain; 7 Niche Science & Technology, Unit 26, Falstaff House, Bardolph Road, Richmond, TW9 2LH, UK; 8 Institute of Diabetes for Older People (IDOP), University of Bedfordshire, Putteridge Bury Campus, Hitchin Road, Luton, Bedfordshire LU2 8LE, UK

**Keywords:** Multi-modal intervention, Frail, Pre-frail, Type 2 diabetes

## Abstract

**Background:**

Diabetes, a highly prevalent, chronic disease, is associated with increasing frailty and functional decline in older people, with concomitant personal, social, and public health implications. We describe the rationale and methods of the multi-modal intervention in diabetes in frailty (MID-Frail) study.

**Methods/Design:**

The MID-Frail study is an open, randomised, multicentre study, with random allocation by clusters (each trial site) to a usual care group or an intervention group. A total of 1,718 subjects will be randomised with each site enrolling on average 14 or 15 subjects. The primary objective of the study is to evaluate, in comparison with usual clinical practice, the effectiveness of a multi-modal intervention (specific clinical targets, education, diet, and resistance training exercise) in frail and pre-frail subjects aged ≥70 years with type 2 diabetes in terms of the difference in function 2 years post-randomisation. Difference in function will be measured by changes in a summary ordinal score on the short physical performance battery (SPPB) of at least one point. Secondary outcomes include daily activities, economic evaluation, and quality of life.

**Discussion:**

The MID-Frail study will provide evidence on the clinical, functional, social, and economic impact of a multi-modal approach in frail and pre-frail older people with type 2 diabetes.

**Trial registration:**

ClinicalTrials.gov: NCT01654341.

## Background

Diabetes has a high prevalence in ageing populations, affecting approximately 20% of people aged 70 years or over. It is anticipated that by 2050 the number of cases of diabetes will have increased by fourfold in people older than 70 years [1]. Diabetes is associated with increasing frailty and functional decline in older people [2]. Frailty is defined as a clinical syndrome in which three or more of the following criteria are present: unintentional weight loss (≥4.5 kg in past year), self-reported exhaustion, weakness (grip strength), slow walking speed, and low physical activity [3].

Diabetes has serious personal and social consequences, and is a significant public health burden in terms of rising health care costs; in Spain, annual direct health care costs have been estimated at 2.5 billion euros [4]. In recent studies of older people, up to 28% of those with diabetes required some help with activities of daily living, compared with 16% of those without the condition [5]. This functional decline can be explained in only half of the cases by the classical complications of the disease, such as coronary artery disease, stroke, and peripheral vascular disease [5]. The worsening in functional status due to deterioration of skeletal muscle, increased co-morbidities, and adverse effects of overmedication associated with diabetes, results in many older frail people becoming more disabled, with an impaired quality of life associated with increased use of health care resources [6-8].

There is a marked lack of intervention studies that aim to reduce functional decline and improve quality of life in older people with diabetes and until relatively recently, most of the clinical guidelines for treating type 2 diabetes were of limited use in these subjects. A focus on improvements in function and well-being may be fundamentally of more clinical benefit in older frail people with diabetes than attention to metabolic control alone [9]. In older adults and frail individuals, resistance training is now considered to be an important component of diabetes management and prevention, mainly through increasing muscle mass, strength, and power [10]. This leads, in turn, to increased mobility and a decreased risk of falling [11]. In addition, resistance training has been proven to improve insulin sensitivity and fasting glycaemia, and to decrease abdominal fat in older people with type 2 diabetes [10]. It has been previously shown that intensive glucose lowering therapy (targeting a glycated haemoglobin level below 6.0%) in patients with type 2 diabetes was associated with a reduction in 5-year non-fatal myocardial infarction but increased 5-year mortality [12,13]. More recently, it was reported that an intensive lifestyle intervention focusing on weight loss in overweight or obese adults with type 2 diabetes led to improvements in glycaemic control, blood pressure, high-density lipoprotein (HDL)-cholesterol, and triglycerides [14]. However, the intervention failed to reduce the incidence of cardiovascular events [15]. These observations provide sufficient motivation for evaluating non-metabolic control as an alternative (and complementary) way of improving clinical outcome.

The long-term impact of diabetes in an ageing population results in substantial health care expenditure. This further emphasises the potential value of an intervention that can prevent or delay the onset of diabetes-associated frailty. The multi-modal intervention in diabetes in frailty (MID-Frail) study focuses on the use of interventions designed to improve functional status and enhance quality of life by acting on the mechanisms involved in frailty and its progression to adverse outcomes [16]. The MID-Frail study brings together 16 partners from seven countries in the European Union (EU) and will run for 4 years, with the intervention lasting 2 years. The primary objective of the MID-Frail study is to evaluate, in comparison with usual clinical practice, the effectiveness of a multi-modal intervention (specific clinical targets, education, diet, and exercise) in frail and pre-frail subjects aged ≥70 years with type 2 diabetes in terms of the difference in function 2 years post-randomisation. Difference in function will be measured by changes in summary ordinal score on the short physical performance battery (SPPB) of at least one point.

## Methods/design

### Subjects

#### Inclusion criteria

Subjects are eligible to enter the study if all of the following apply:

1) The subject is willing and able to give written informed consent for participation in the study.

2) The subject is aged 70 years or older, with a diagnosis of type 2 diabetes for at least 2 years.

3) The subject fulfils the Fried’s criteria for frail or pre-frail individuals (Table 1).

4) The caregiver has agreed to participate in the study and to give informed consent for participation in the carer burden part of the study. If the caregiver does not agree, the subject may continue to participate in the study, but information about caregiver burden will not be collected.

**Table 1:** Fried’s criteria for study inclusion

**Criterion**	**Definition**
1. Weight loss	Unintentional weight loss of 4.5 kg during the past year
2. Exhaustion	Using the responses (yes/no) to two statements on the CES-D scale
3. Physical activity	Is the weekly physical activity of the subject lower or equal to (yes/no): men: <383 kcal per week (walking: <2.5 hours per week) women: <270 kcal per week (walking <2 hours per week)
4. Slowness	Assessed by walk time and stratified by gender and height
5. Weakness	Assessed by grip strength and stratified by gender and BMI

Frailty is indicated by satisfying three or more of the criteria; pre-frailty is indicated by satisfying one or two of the criteria [3]. BMI, body mass index; CES-D, Center for Epidemiologic Studies Depression.

Fried’s criteria for frailty were chosen as they are easy to implement and allow the selection of subjects who are not dependent (moderately or highly). In addition, these criteria should avoid selection bias and prevent the enrolment of a high percentage of subjects with disabilities at baseline.

#### Exclusion criteria

Subjects cannot enter the study if any of the following apply:

**Figure 1:**
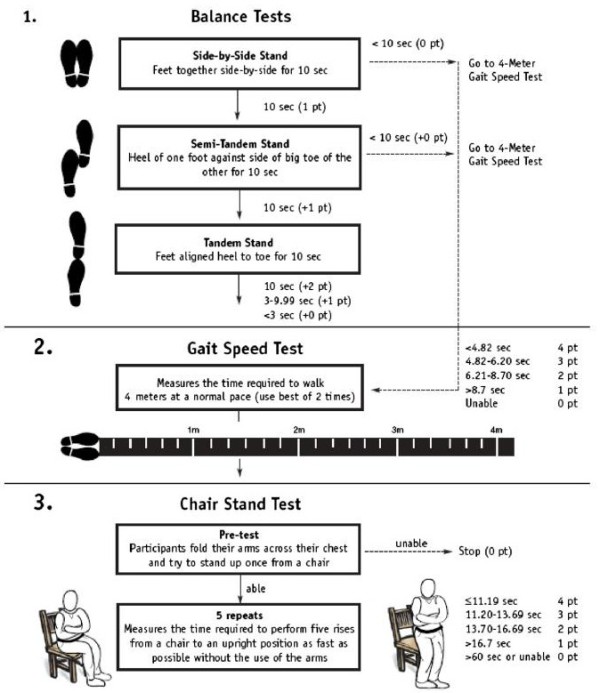
**The short physical performance battery (SPPB)[**[21]**].** Reprinted from Journal of Biomechanics, Vol. 45, Riskowski JL, Hagedorn TJ, Dufour AB and Hannan MT, Functional foot symmetry and its relation to lower extremity physical performance in older adults: the Framingham Foot Study, pp. 1796–1802, Copyright 2012, with permission from Elsevier.

1) Barthel score lower than 60 points [17].

2) Inability to complete the SPPB (total score = 0) (Figure 1) [18].

3) Mini Mental State Examination (MMSE) score of less than 20 points [19].

4) Subject is unwilling or unable to consent or unable to participate safely in the intervention programme.

5) Previous history of myocardial infarction within 6 months, unstable angina, or congestive heart failure at stage III to IV of the New York Heart Association (NYHA) classification [20].

6) The subject is clinically unstable in the clinical judgment of the investigator.

7) Terminal illness (life expectancy <6 months).

8) Any other condition that, in the clinical judgment of the investigator, means that it would not be in the subject’s best interests to enter the study.

9) Concurrent participation in a clinical trial or any other investigational study.

To date, the study has been approved by 16 institutional ethics committees in Spain (see further details in Additional file 1). The study will be carried out in accordance with Good Clinical Practice, applicable local regulatory requirements, and the guiding principles of the Declaration of Helsinki.

### Study design

**Figure 2:**
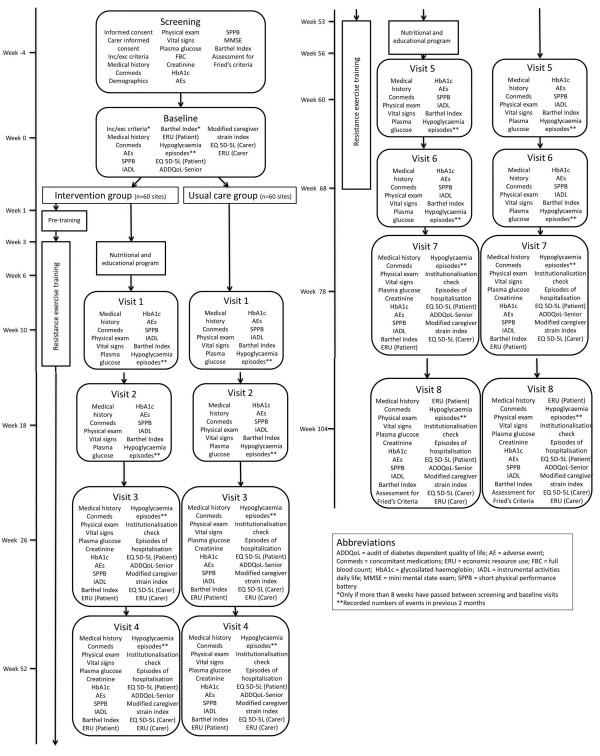
Detailed study flowchart.

This is an open, randomised, multicentre study, with random allocation by clusters (each trial site) to a usual care group or an intervention group (Figure 2). Each site will enroll on average 14 or 15 subjects. National research centres in Belgium, Czech Republic, Italy, and Germany (one each) and in France, United Kingdom, and Spain (two each) will each be responsible for 11 or 12 sites. Each site will be monitored to ensure full adherence to the study protocol and the integrity of the data collected. An adaptive monitoring strategy has been adopted in which some sites will be monitored remotely. The study will evaluate the intervention through the difference in functioning between intervention and usual care groups; an economic evaluation of the intervention and of quality of life will also be carried out.

#### Randomisation

Before randomisation, each trial site will identify and make a list of 50 to 75 potential subjects. Identifying individuals for recruitment before randomisation will help to avoid subject selection bias [22]. Once this list is complete, the investigator will have 1 month to obtain signed informed consent from at least seven subjects (roughly half of the site target size) on that list. Then, the site will be randomised and exercise machines delivered. Randomisation will be performed centrally by onmedic (Barcelona, Spain) in collaboration with Cardiff University (Cardiff, UK).

#### Intervention group

The study intervention consists of a multi-modal programme comprising glycaemia and blood pressure monitoring to reach pre-defined targets, a 16-week resistance exercise programme based on leg press and leg extension exercises (Additional file 2), and a nutritional and educational programme. The exercise and the nutritional and educational programmes will be run at the study site at the beginning of year 1 and repeated at the beginning of year 2. All subjects in this intervention group will follow the multi-modal programme.

##### Glycaemia and blood pressure monitoring

The targets for glycaemia and blood pressure control will be pre-defined; however, the treatment required to achieve them will not be pre-defined. Treatment protocols at each site will be used to ensure that target values are reached within 6 months of enrolment. There will be no target for cholesterol or its fractions and there are no specific recommendations for the use of aspirin. The target for glycaemia will be: optimal glycated haemoglobin in the range of 7 to 8% (53 to 64 mmol/mol); the target for blood pressure will be <150/90 mmHg.

##### Exercise programme

Subjects will undergo 2 weeks of pre-training assessment before the exercise programme begins. They will be familiarised with the exercises through several submaximal and maximal performances, using one-repetition maximum (1RM), and baseline measurements will be taken. In all tests of exercise performance, subjects will be encouraged verbally to perform each test action as forcefully and as rapidly as possible.

##### Exercise methodology

The exercise programme used in the MID-Frail study will be similar to one described previously [10,23]. The subjects will be asked to report to the training facility on 2 days each week; each session will last from 20 to 30 minutes. Training sessions will be separated by a minimum of 2 days. Subjects will undertake supervised resistance exercise for 16 weeks starting at week 2 (baseline) and the assessments carried out at baseline will be repeated in week 6, week 10, and week 18. Each training session will include two exercises for the leg extensor and knee extensor muscles. Only resistance machines will be used throughout the training period (Exercycle, Exercycle S.L., Alava, Spain). For the first 8 weeks of the training period, subjects will train with loads of 40 to 60% of the individual 1RM, 8 to 12 repetitions per set, and 2 to 3 sets. For the last 8 weeks of the training period, the loads will be 70 to 80% of the maximum, 4 to 6 repetitions per set (higher loads), and 3 to 4 sets.

##### Nutritional and educational programme

The MID-Frail nutritional and educational programme is aimed specifically at older people with diabetes and is derived from a published programme for this particular population [24]. The programme consists of sessions that aim to increase the subject’s knowledge and understanding of diabetes, to develop practical skills in diabetes self-management, and to enhance the likelihood of improved and safe control of glycaemia [25].

Each subject will undergo pre-trial assessment of nutritional status and any requirement for weight reduction or weight gain and the need for diet modification. Thereafter, the intervention consists of clinician-moderated sessions in a clinic or general practitioner surgery with small groups of four to eight subjects. Seven separate 45-minute sessions will be delivered, with two each week for 3 to 4 weeks; the sessions will be held on the same day as the exercise sessions.

#### Usual care group

Subjects in the usual care group will receive the routine care a subject with diabetes would normally be expected to receive from his/her local health care system, including his/her general practitioner.

### Outcome measures

The primary outcome measure is the incidence of functional impairment as measured by the difference in function after 2 years of follow-up between intervention and usual care groups, assessed by changes in summary ordinal score on the SPPB test ≥1. Data from a previous study have shown that one point is the minimum change in the SPPB test that can be considered clinically significant [26]. Secondary outcome measures are listed in Table 2.

**Table 2:** Primary and secondary outcome measures

**Outcome measure**	**Definition**
Primary	The difference in function after 2 years of follow-up between intervention and usual care groups, according to changes in summary ordinal score on the SPPB test ≥1 [26].
Secondary	a) Barthel ADL index [13] and b) Lawton IADL scale [27].
	c) Quality of life, as measured by using the EuroQoL index, EQ-5D-5 L [28].
	d) Economic costs/health care expenditure due to diabetes and its impact on disability and quality of life, using an economic model embracing the direct health-related costs (in-subject, out-subject, pharmaceutical), formal care costs (home care, respite care, day centres), and the informal care costs (carer).
	e) Episodes of symptomatic hypoglycaemia (that is, a recorded blood sugar <4 mmol/L, or symptoms or signs attributed to low blood sugar and responding to appropriate treatment).
	f) Episodes of hospital admission (that is, any admission involving an overnight stay).
	g) Episodes of permanent institutionalisation (that is, permanent move to any care setting other than the subject’s own home, where paid staff are available to provide care if needed at any time during the day or night).
	h) Burden of the carer, as assessed by the Modified Caregiver Strain Index (MCSI) [29].
	i) Mortality.

ADL, activities of daily living; IADL, instrumental activities of daily living; MCSI, Modified Caregiver Strain Index; SPPB, short physical performance battery.

#### Economic assessments

The main aim of the economic assessment is to estimate the incremental cost-effectiveness ratio of the multi-modal intervention in frail and pre-frail subjects aged ≥70 years with type 2 diabetes in comparison with usual best clinical practice. Three types of cost will be assessed through a comparative analysis of the alternative courses of action in terms of both their financial costs and their health outcomes: 1) direct health care costs, these costs are related to in-subject and out-subject treatment, and medicines; 2) formal care (mainly social services) costs, this includes those services that involve public or private funding, including the use of a day centre, nursing home, residential care, home support services, and personal alarm system; and 3) informal care costs, informal support includes the different types of non-paid support provided by relatives and friends as a result of the subject’s disability.

### Safety assessments

All co-existing diseases or conditions will be treated in accordance with prevailing medical practice. All medications (prescription and over-the-counter) that started before screening may be continued during the study and will be recorded as concomitant therapy on the electronic case report form (eCRF). Medications for any conditions that may arise after screening, or for worsening of an existing condition, will be allowed and recorded on the eCRF; the condition will be reported as an adverse event. Standard safety data on adverse drug reactions will be collected and reported.

### Power and sample size

The sample size per group (intervention and usual care group) will be 859 subjects. This sample size has been calculated according to the following assumptions: a yearly incidence of functional impairment in frail/pre-frail subjects (main variable) of 30% [30], accumulated incidence of functional impairment in 2 years of intervention of 51% (assuming a constant 30% incidence rate year on year), an intervention effect size of 20%, a z statistic to compare the proportions of dichotomous variables (as the statistical test), a two-tailed α = 0.05, a two-tailed 1-β = 0.8, an intra-cluster coefficient correlation of 0.05, an average cluster size of 15, and a coefficient of variation cluster size of 0.25 [31,32]. Assuming a 20% loss of subjects during follow-up, the final sample size will be 1,718 subjects.

### Statistical analysis

The primary analysis will compare the odds of developing functional impairment in the intervention group with the usual care group, controlling for baseline function, using a hierarchical logistic regression model with subjects nested within trial sites. This will be an intention-to-treat analysis controlling for subject characteristics, such as subject age, gender, and comorbidities. The groups will be compared in terms of odds ratios and relative risks.

Secondary outcome variables include: 1) quality of life as measured by the quality of life measurement tool EQ-5D-5 L (EuroQoL, Rotterdam, Netherlands), which will be used in the economic evaluation; 2) hospital admission, mortality, and permanent institutionalisation, which will be investigated using hierarchical logistic regression models, and the results summarised using odds ratios and relative risks. If there are multiple outcomes per subject (for example hospital admission), Poisson regression may be used. Hierarchical survival analysis may also be used to model the time to first event; 3) carer burden and quality of life, which will be addressed using hierarchical regression models; and 4) validation of the Audit of Diabetes-Dependent Quality of Life (ADDQoL) senior scale [33], including measuring internal consistency (using Cronbach’s alpha) and establishing convergent and divergent validity (by comparing subject characteristics expected and not expected to be related with ADDQoL senior and ADDQoL senior scores). Floor and ceiling effects will be investigated. Correlation analysis will be conducted to identify whether the ADDQoL senior scale correlates with the other measures of function used in this study. Test re-test reliability will also be estimated (the ADDQoL senior will be administered at weeks 0, 26, 52, 78, and 104).

All analyses, except for the economic assessment, will be run by team members at Cardiff University in collaboration with the study and sub-study teams. The economic analysis will be performed by team members from Universidad Castilla-La Mancha (Ciudad Real, Spain). Standard imputation methods (for example mean value imputation, last observation carried forward) will be used to impute missing data depending on the pattern of missing data. Dropout due to death is a possibility in this study population. A sensitivity analysis will be run in which dropouts due to death will be assigned the minimum function score. Joint modelling of function and dropout due to death will also be explored. A causal adjusted complier analysis will be used to estimate the treatment effect observed in those subjects who complied with the intervention. Survival analysis naturally deals with censoring, which will help minimise the effect of dropout in the secondary analyses. No interim analysis is planned and is not accounted for in the sample size calculation. An ethics independent external advisory committee will be formed and will meet at least three times during the course of the study to evaluate its progress, the safety data, the critical efficacy endpoints, and will make any recommendations to the sponsor and the steering committee whether to continue, modify, or stop the study.

## Discussion

The rapid increase in the number of older people combined with the high prevalence of diabetes in ageing populations has created an urgent need for effective interventions to prevent or delay the onset of frailty and functional decline in older people. The MID-Frail study will address the lack of intervention studies in older people with diabetes by examining a comprehensive, multi-modal intervention designed to reduce functional decline and improve quality of life.

It is estimated that if the MID-Frail intervention is successful in reducing disability and functional decline, 700,000 fewer cases of disability will be reported every year, accompanied by health care savings of more than 3 billion euros per year across the EU. The MID-Frail nutritional and educational programme is intended to prevent loss of muscle protein and ensure optimal nutritional status, to minimise the risk of hypoglycaemia, and assist in maintaining functional status. Investigators will be guided on how to prepare each session and how to maximise participants’ enjoyment and benefits through interaction. Individual nutritional goals will be set for each subject and there will be a focus on behavioural change. The specific dietary needs of older people will be emphasised and the programme will be tailored in each country to take into account the influence local social and cultural norms have on diet.

The study will be randomised using the cluster method; this is necessary because the education and the exercise programmes will be done in groups, and this will also avoid or control for contamination bias. The MID-Frail study will run for 4 years with a 2-year intervention. We will capitalise on the long duration of the study and the large number of subjects involved by carrying out several sub-studies, which will run concurrently with the main study and will evaluate complementary research questions: 1) the characteristics of skeletal muscle and adjacent tissues will be investigated by sonoelastography at the beginning and at the end of the intervention (Sartrain Sub-study); 2) exercise-induced, short- and long-term changes in muscle power output, balance, and gait will be studied as mediators of the final response on function (MID-POW Sub-study); 3) kinetic and kinematic movement data will be analysed (Sensole Sub-study); 4) the metabolic profile of frail/sarcopenic older subjects with diabetes will be characterised and changes in these parameters following the intervention will be profiled (MetaboFrail Sub-study); and 5) polymorphisms of three genes (*Pro259Arg-TCN2* gene; *ACE I/D-ACE* gene, and *e2/e3/e4-Apo E* gene) will be determined to establish their predictive value for the development of disability and response to treatment (GeneFrail Sub-study).

The MID-Frail study will provide evidence on the clinical, functional, social, and economic impact of a multi-modal approach in frail and pre-frail older people with diabetes.

## Trial status

The trial is currently being set up.

## Abbreviations

1RM: One-repetition maximum; ADDQoL: Audit of Diabetes-Dependent Quality of Life; ADL: Activities of daily living; BMI: Body mass index; CES-D: Center for Epidemiologic Studies Depression; eCRF: Electronic case report form; EU: European Union; HDL: High-density lipoprotein; IADL: Instrumental activities of daily living; MCSI: Modified Caregiver Strain Index; MID-Frail: Multi-modal intervention in diabetes in frailty; MMSE: Mini Mental State Examination; NYHA: New York Heart Association; SPPB: Short physical performance battery.

## Competing interests

The authors declare that they have no competing interests.

## Authors’ contributions

LRM participated in the conception, design, and writing of the study. AJB outlined the statistical analysis and performed the sample size calculations. MK provided input on the statistical analysis. AZ designed the educational intervention. MI designed the exercise programme. OL participated in the design of the study. TCH participated in the design and writing of the study. AJS participated in the conception, design, and writing of the study. All authors read and approved the final manuscript.

## Supplementary Material

Additional file 1 Details of institutional ethics committees. Click here for file

Additional file 2 Leg exercises. Click here for file
