# Supplementary material for: An evaluation of the effectiveness of a multi-modal intervention in frail and pre-frail older people with type 2 diabetes - the MID-Frail study: study protocol for a randomised controlled trial
Source: Trials. 2014 Jan 24;15:34. doi: 10.1186/1745-6215-15-34 (PMC3917538; doi:10.1186/1745-6215-15-34)
Supplement: Additional file 1 — Details of institutional ethics committees. [file 1745-6215-15-34-S1.docx]

Additional File 1. Details of the Institutional Ethics

| Name of the Ethics Committee, Address |
| --- |
| CEICA, Departamento de Salud y Consumo del Gobierno de Aragón, Avda Gómez Laguna 25, Zaragoza, 50009 - Zaragoza |
| IDIAP Jordi Gol, Av. Gran Via de les Cortes Catalanes, 587 Atic, 08007 - Barcelona |
| CEIC Hospital Clínico San Carlos, Doctor Martín Lagos, s/n, Madrid 28040 - Madrid |
| Servicio Canario de la Salud, Hospital Universitario de Canarias, Ofra, s/n, La Cuesta, 38320 La Laguna - Santa Cruz de Tenerife |
| Complejo Hospitalario Universitario de Albacete, Hnos. Falcó, 37, 02006 - Albacete |
| CEIC, Hospital Universitario de La Paz, Pº de la Castellana, 261, 28046 - Madrid |
| Hospital Universitario de Getafe, Autovía de Toledo 28905 Getafe - Madrid |
| CEI Granada, Hospital Universitario Virgen de las Nieves, Avda. Fuerzas Armadas, 2, 18014 - Granada |
| Comité Ético de Investigación Clínica de la Fundacion Jimenez Diaz, Avda. De los Reyes Católicos, 2, 28040 - Madrid |
| Comité Ético de Investigación Clínica del Hospital Universitario de la Princesa, C/ Diego de León 62, 28006 – Madrid |
| Comisión de Investigación Departamento de Salud de la Ribera, Carretera de Corbera km 1  46600 - Alzira- Valencia |
| Comité de Ética de la Investigación Málaga Nordeste, Hospital Regional Universitario Carlos Haya, Avda. Carlos Haya, s/n, 29010 - Málaga |
| Comité de Ética de la Investigación Clínica Regional del Principado de Asturias, C/ Calestino Villamil, s/n, 33006 - Oviedo |
| Hospital Universitari de Girona, Avinguda de França, s/n, 17007 – Girona |
| Comité Ético de Investigación Clínica del area de Salud de Salamanca, Hospital Universitario de Salamanca, Paseo de San Vicente, 58–182, 37007 – Salamanca |
| Comité Ético de Investigación Clínica de Galicia, Edificio Administrativo de San Lázaro, 15781 - Santiago |
