# Supplementary material for: An evaluation of the effectiveness of a multi-modal intervention in frail and pre-frail older people with type 2 diabetes - the MID-Frail study: study protocol for a randomised controlled trial
Source: Trials. 2014 Jan 24;15:34. doi: 10.1186/1745-6215-15-34 (PMC3917538; doi:10.1186/1745-6215-15-34)

Additional File 2. Leg press (A) and leg extension (B) exercises that comprise the exercise element of the MID-Frail intervention.


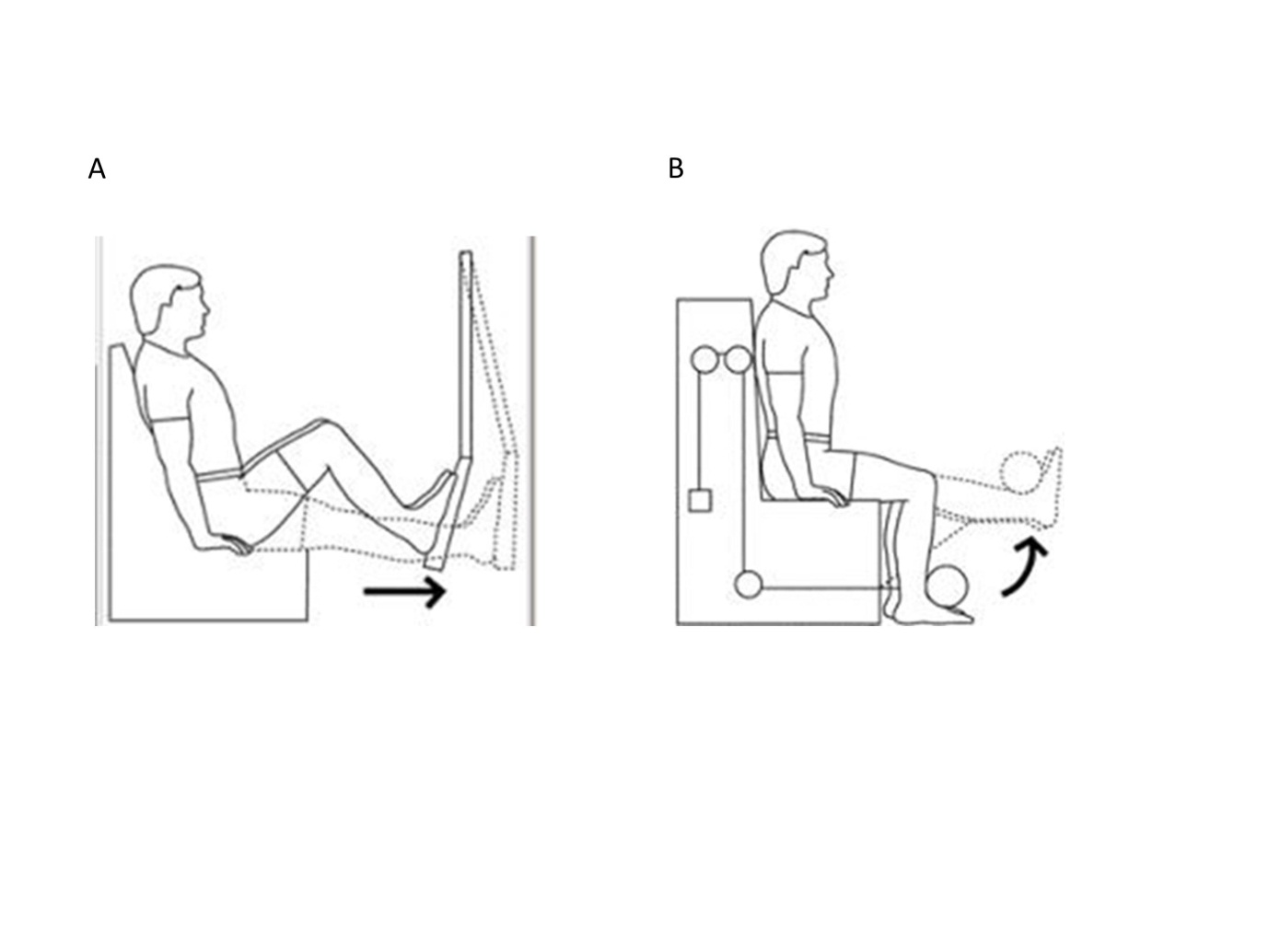

Supplement: Additional file 2 — Leg exercises. [file 1745-6215-15-34-S2.docx]
